# Responsive changes of DNA methylation in wheat (*Triticum aestivum*) under water deficit

**DOI:** 10.1038/s41598-020-64660-7

**Published:** 2020-05-13

**Authors:** Hongying Duan, Jingyun Li, Yanqiu Zhu, Wenjing Jia, Huihui Wang, Lina Jiang, Yanqing Zhou

**Affiliations:** College of Life Sciences, Henan Normal University, Xinxiang, 453007 China

**Keywords:** Abiotic, Drought

## Abstract

DNA methylation plays an important role in the growth and development of plant, and would change under different environments. In this study, 5-methyl cytosine (5mC) content and methylation level exhibited tissue specificity in genomic DNA of wheat seedling, and increased significantly in leaf along with the increase of water deficit, which was especially significant in leaf of wheat AK58. Full-methylation might dominate in genomic DNA of wheat seedling, the increase of full-methylation level under water deficit was significantly higher than that of hemi-methylation level. Under water deficit, DNA methylation of wheat seedling showed significant polymorphism, this polymorphism was always higher in root, especially was higher in root of wheat AK58. Further analysis appeared that changes of DNA methylation in wheat seedling took methylation as principle and demethylation as supplement under water deficit. Therefore, under water deficit, the degree, level and polymorphism of DNA methylation in wheat seedling showed tissue specificity and species specificity, and were higher in wheat AK58 compared with those of wheat XM13, perhaps wheat AK58 could more rapidly respond to water deficit by changes of DNA methylation, which would contribute to reveal molecular mechanism of wheat adapting to water deficit.

## Introduction

Growth and development of plant are often influenced by environment, yet some studies indicated that plant could rapidly respond to the change of environment by epigenetic modification^1^. As one important mode of epigenetic modifications, DNA methylation could regulate gene expression by changing chromatin structure, DNA conformation, DNA stability, DNA-protein interaction and so on^2^. In nuclear genome of plant, about 20–30% cytosines are methylated, and levels of DNA methylation are different in all kinds of tissues, organs or stages^3–5^. If DNA methylation is insufficient or increases in plant, growth and phenotype of plant might be aberrant^6–8^, for example, *Arabidopsis thaliana* would exhibit dwarf plant, smaller leaf, clump growth or maturity decline because of the reduction of DNA methylation, and these aberrant traits may be inherited to filial generation^9^. Manning *et al*. found that DNA hyper-methylation of Cnr point in *Tomato* would inhibit the maturation of fruit and cause appearance variation of fruit, such as colorless fruit, pericarp absence, etc^6^.

Furthermore, level and status of DNA methylation might change under stress conditions, such as salt^10–12^, drought^13–15^, low temperature^16^, heavy metal^17^, pathogen^18^, and so on^19^. Water deficit could lead to hyper-methylation in *Pea* and methylation level of second C in CCGG sequence increases by 40%^20^, low temperature would cause methylation and demethylation at some points of CCGG sequence in *Oryza sativa*^21^. Under salt stress, methylation level of cytosine in CCGG sequence would increase by 0.2–17.6% in *Rape seed*^22^, and methylation level in *Manioc* would increase significantly^23^. Tang *et al*. also found that drought might cause methylation level decrease by 10% in *Ryegrass*^13^. Therefore, methylation state of plant could be influenced by environment, and plant can respond to different environments by the change of DNA methylation.

Wheat belongs to one of important crops in the world, the quality and yield of wheat are seriously influenced by drought, some studies also showed that DNA methylation of plant would change under drought stress^13–15^, yet the relationship between DNA methylation and drought-tolerant mechanism is unclear in wheat. In order to study the response of DNA methylation to water deficit, common wheat genotype XinMai 13 (XM13) and resistant wheat genotype AiKang 58 (AK58) were selected as experimental materials in this study, the change of DNA methylation in wheat seedling under water deficit was analyzed with High Performance Liquid Chromatography (HPLC) and Methylation Sensitive Amplification Polymorphism (MSAP), which would provide reference to reveal molecular mechanism of wheat adapting to water deficit from the perspective of epigenetics, and should be significant to explain drought-tolerant mechanism of wheat.

## Results

### The content of 5mC in wheat seedlings

**Figure 1:**
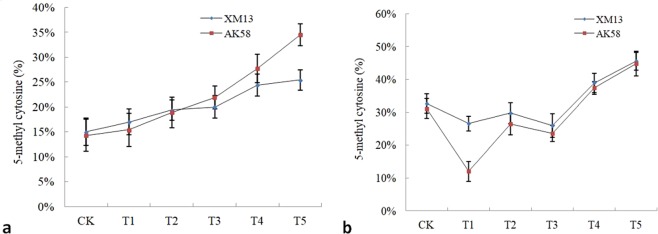
Content of 5mC in wheat seedlings quantified by HPLC. (**a**) 5mC content in leaf of wheat XM13 or wheat AK58, (**b**) 5mC content in root of wheat XM13 or wheat AK58. CK, T1, T2, T3, T4 or T5 represented the treatment with 0.00, −0.05, −0.15, −0.30, −0.50, −0.75 MPa PEG_6000_ solution, respectively. The error bar was standard error of mean, the different lowercase letters above bars separately indicated significant difference of DNA methylation among treatments of two wheat cultivars (P < 0.05).

As shown in Fig. 1(a), 5mC content in leaf of wheat seedlings was progressively higher along with the increase of water deficit. At the osmotic potential below −0.15 MPa, 5mC content in leaf of wheat AK58 was significantly higher than the control (*P* < 0.05), and the increase extent of 5mC content in leaf of wheat XM13 was obviously less compared with that of wheat AK58 (Fig. 1a). Compared to the control, 5mC content in root of wheat seedlings was significantly lower above −0.30 MPa (Fig. 1b), especially was only 12.0% in root of wheat AK58 as treated with −0.05 MPa. However, at the osmotic potential below −0.50 MPa, 5mC content in root was obviously higher than the control (*P* < 0.05), and increased significantly along with the increase of water deficit.

Furthermore, compared with that in root of wheat XM13, as treated with −0.05 MPa PEG_6000_, the decline extent of 5mC content was obviously higher in root of wheat AK58, but decline or increase extent of 5mC content in root of two wheat cultivars was similar at the osmotic potential below −0.15 MPa (Fig. 1b). In addition, along with the increase of water deficit, 5mC content in leaf and root presented different change trend, increased significantly in leaf (Fig. 1), especially was much higher than the control at −0.75 Mpa (Fig. 1a). However, compared to the control, 5mC content in root was lower under mild water deficit (above −0.30 MPa PEG_6000_), and was significantly higher under severe water deficit (below −0.50 MPa PEG_6000_) (Fig. 1b).

### Level of DNA methylation in wheat seedlings

As listed in Table 1, under water deficit, methylation level increased significantly in leaf, the increase extent was similar in two wheat cultivars and was up to about 8–10% below −0.50 MPa, yet methylation level in leaf of wheat AK58 was higher compared with that of wheat XM13. The increase extent of full-methylation level was also similar in two wheat cultivars, and was significantly higher compared with hemi-methylation level, however, at the osmotic potential below −0.50 MPa, hemi-methylation level increased by 4–5% in leaf of wheat AK58 and was higher than that in leaf of wheat XM13 (Table 1).

**Table 1:** Methylation level in leaf of wheat seedlings determined by MSAP.

	Methylation level (%)		Hemi-methylation level (%)		Full-methylation level (%)	
	Wheat XM13	Wheat AK58	Wheat XM13	Wheat AK58	Wheat XM13	Wheat AK58
CK	18.61d	20.74c	4.38b	3.68bc	14.23d	17.06bc
T1	19.66cd	21.24c	3.45bc	2.94c	16.21c	18.30b
T2	21.87c	23.03bc	4.18b	4.42b	17.69bc	18.61b
T3	24.54bc	24.71bc	4.60b	4.65b	19.94ab	20.06ab
T4	26.98b	28.80a	6.81ab	7.73a	20.16ab	21.07a
T5	28.42ab	30.62a	7.49a	8.61a	20.93a	22.01a

CK, T1, T2, T3, T4 and T5 represented wheat seedlings under different osmotic potentials of PEG_6000_ solution with 0.00, −0.05, −0.15, −0.30, −0.50 or −0.75 MPa, respectively. The different lowercase letters in the same column denoted the significant difference among treatments of two wheat cultivars (P < 0.05).

As shown in Table 2, methylation level increased significantly in root of wheat AK58 under water deficit (*P* < 0.05), and was up to 31.1–33.8% at the osmotic potential below −0.50 MPa. Compared with the control, methylation level in root of wheat XM13 was lower at the osmotic potential above −0.30 MPa, but increased to 31.1–33.1% at the osmotic potential below −0.50 MPa (*P* < 0.05), furthermore hemi-methylation level in root of wheat XM13 increased significantly (*P* < 0.05), yet its full-methylation level decreased except the treatment of −0.75 MPa. Different from that of wheat XM13, full-methylation level in root of wheat AK58 showed significantly increase (*P* < 0.05), but its hemi-methylation level had no significant changes except the treatment of −0.75 MPa. Thus, the level of DNA methylation increased in root of wheat AK58 under water deficit, but only increased in root of wheat XM13 under severe water deficit.

**Table 2:** Methylation level in root of wheat seedlings determined by MSAP.

	Methylation level (%)		Hemi-methylation level (%)		Full-methylation level (%)	
	Wheat XM13	Wheat AK58	Wheat XM13	Wheat AK58	Wheat XM13	Wheat AK58
CK	28.95c	26.17d	5.85c	7.79b	23.10a	18.38c
T1	26.84cd	27.13cd	6.78bc	6.31bc	20.06b	20.82b
T2	27.58bc	28.61bc	7.69b	7.22b	19.89b	21.39ab
T3	27.96bc	29.25bc	8.06ab	7.24b	19.90b	22.01a
T4	31.10ab	31.19b	8.61a	7.62b	22.49a	23.57a
T5	33.11a	33.85a	9.24a	10.24a	23.87a	23.61a

CK, T1, T2, T3, T4 and T5 represented wheat seedlings under different osmotic potentials of PEG_6000_ solution with 0.00, −0.05, −0.15, −0.30, −0.50 or −0.75 MPa, respectively. The different lowercase letters in the same column denoted the significant difference among treatments of two wheat cultivars (P < 0.05).

### Status of DNA methylation in wheat seedlings

**Figure 2:**
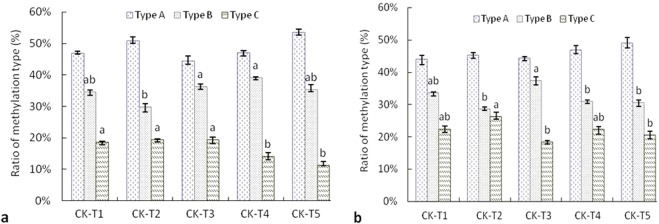
Methylation status in leaf of wheat seedlings under water deficit. (**a,b**) represented the ratio of methylation type in leaf of wheat XM13 or wheat AK58, respectively. CK, T1, T2, T3, T4 and T5 indicated wheat seedlings under different osmotic potentials of PEG_6000_ solution with 0.00, −0.05, −0.15, −0.30, −0.50 or −0.75 MPa, respectively. Type A: methylation monomorphism, ratio of Type A (%) = (band A2 + band A3)/(total methylation bands) ×100; Type B: methylation type, ratio of Type B (%) = (band B1 + band B2 + band B3 + band B4 + band B5)/(total methylation bands) × 100; Type C: demethylation type, ratio of Type C (%) = (band C1 + band C2 + band C3)/(total methylation bands) × 100; Total methylation bands = bands of Type A + bands of Type B + bands of Type C. The error bar was standard error of mean, the different lowercase letters above bars represented the significant difference among treatments of the same methylation type (P < 0.05).

In this study, methylation pattern had significant change in leaf under water deficit (Table 3), compared with that of wheat XM13, the change of methylation pattern was higher in leaf of wheat AK58 as treated with the same osmotic potential of PEG_6000_, particularly was significant at the osmotic potential −0.15 to −0.05 MPa (*P* < 0.05). As shown in Fig. 2, the ratio of methylation and demethylation was different in leaf of two wheat cultivars under water deficit (Fig. 2), methylation polymorphism was significantly higher in leaf of wheat XM13 at the osmotic potential below −0.50 MPa (*P* < 0.05), but demethylation polymorphism was significantly higher in leaf of wheat AK58 (except for −0.30 MPa).

**Table 3:** Change of methylation pattern in leaf of wheat seedlings under water deficit.

Type of methylation pattern	Number of DNA methylation band									
	Wheat XM13					Wheat AK58				
	CK-T1	CK-T2	CK-T3	CK-T4	CK-T5	CK-T1	CK-T2	CK-T3	CK-T4	CK-T5
A1	205	211	219	219	223	199	203	209	223	228
A2	10	12	14	15	19	11	12	15	14	19
A3	43	46	46	49	52	48	45	48	51	55
B1	2	5	3	7	5	4	6	9	2	4
B2	13	5	11	9	10	11	4	16	14	12
B3	15	12	16	23	21	18	19	11	10	9
B4	1	0	2	5	4	3	2	6	5	8
B5	8	12	17	9	7	9	5	11	12	13
C1	14	16	13	12	12	16	17	9	14	15
C2	4	2	4	0	1	3	7	9	10	11
C3	0	1	0	0	1	2	3	0	4	1
C4	3	3	9	7	1	9	6	8	3	4
Methylation polymorphism (%)	18.87c	17.23cd	21.19ab	20.28b	17.42cd	22.52a	20.97ab	22.51a	20.44b	20.32b

The type of DNA methylation pattern was listed as in Table S3, A1-C4 represented change of methylation pattern in leaf under water deficit as compared to the control (CK). CK, T1, T2, T3, T4 and T5 indicated wheat seedlings under different osmotic potentials of PEG_6000_ solution with 0.00, −0.05, −0.15, −0.30, −0.50 or −0.75 MPa, respectively. The different lowercase letters in the row indicated the significant difference among treatments of two wheat cultivars (P < 0.05).

**Figure 3:**
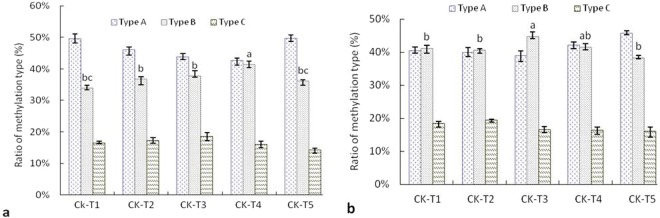
Methylation status in root of wheat seedlings under water deficit. (**a,b**) represented the ratio of methylation type in root of wheat XM13 or wheat AK58, respectively. CK, T1, T2, T3, T4 and T5 indicated wheat seedlings under different osmotic potentials of PEG_6000_ solution with 0.00, −0.05, −0.15, −0.30, −0.50 or −0.75 MPa, respectively. Type A: methylation monomorphism, ratio of Type A (%) = (band A2 + band A3)/(total methylation bands) ×100; Type B: methylation type, ratio of Type B (%) = (band B1 + band B2 + band B3 + band B4 + band B5)/(total methylation bands) × 100; Type C: demethylation type, ratio of Type C (%) = (band C1 + band C2 + band C3)/(total methylation bands) × 100; Total methylation bands = bands of Type A + bands of Type B + bands of Type C. The error bar was standard error of mean, the different lowercase letters above bars represented significant difference among treatments of the same methylation type (P < 0.05).

As listed in Table 4, methylation pattern in root also had significant change under water deficit, compared with that of wheat XM13, the change of methylation pattern was significantly higher in root of wheat AK58 at −0.05 MPa (*P* < 0.05). As shown in Fig. 3, when treated with the same osmotic potential of PEG_6000_, methylation polymorphism in root of wheat AK58 was higher than that of wheat XM13, especially at the osmotic potential above −0.30 MPa (*P* < 0.05), demethylation polymorphism was also higher in root of wheat AK58 (except for −0.30 MPa), but was lower than its methylation polymorphism.

**Table 4:** Change of methylation pattern in root of wheat seedlings under water deficit.

Type of methylation pattern	Number of DNA methylation band									
	Wheat XM13					Wheat AK58				
	CK-T1	CK-T2	CK-T3	CK-T4	CK-T5	CK-T1	CK-T2	CK-T3	CK-T4	CK-T5
A1	195	201	219	228	228	209	211	219	228	231
A2	14	15	15	12	21	13	14	9	18	27
A3	46	49	51	55	56	51	54	52	49	59
B1	0	2	9	4	7	6	9	10	4	9
B2	19	15	17	11	15	17	13	9	13	17
B3	12	18	11	28	23	26	28	27	20	18
B4	0	0	1	3	1	3	5	5	7	9
B5	10	16	19	19	10	13	14	19	22	19
C1	14	16	13	12	12	16	17	9	14	15
C2	1	0	5	1	0	0	0	3	2	0
C3	1	2	0	0	0	1	2	1	1	5
C4	4	6	10	12	10	12	14	13	9	10
Methylation polymorphism (%)	19.30de	22.1c	22.06c	23.38bc	20.37d	25.61ab	26.77a	25.53ab	23.77bc	24.34b

The type of DNA methylation pattern was listed as in Table S3, A1-C4 represented change of methylation pattern in root under water deficit as compared to the control (CK). CK, T1, T2, T3, T4 and T5 indicated wheat seedlings under different osmotic potentials of PEG_6000_ solution with 0.00, −0.05, −0.15, −0.30, −0.50 or −0.75 MPa, respectively. The different lowercase letters in the row indicated the significant difference among treatments of two wheat cultivars (P < 0.05).

In addition, methylation polymorphism in root was higher than that in leaf under water deficit, and was higher in leaf or root of wheat AK58 compared with that of wheat XM13 (Tables 3 and 4), indicating that methylation polymorphism of wheat seedlings had tissue specificity and species specificity under water deficit, and higher methylation polymorphism in leaf or root of wheat AK58 may be related to the higher level of DNA methylation. Furthermore, under water deficit, the change of methylation pattern in leaf or root might take methylation as principle and demethylation as supplement, compared with that in root of wheat AK58, demethylation polymorphism in leaf of wheat AK58 was significantly higher (*P* < 0.05), yet methylation polymorphism was significantly lower in leaf of wheat AK58 (*P* < 0.05).

## Discussions

Drought is one of serious abiotic stresses, and could affect the growth and development of plant. However, plant would exhibit corresponding adaption under drought stress^24,25^, and plant usually responds to drought stress by regulating the expression of stress-responsive gene^26,27^. DNA methylation is important to regulate gene expression^28,29^, and is influenced by development stage, physiological status, environment and other factors^4,5,18,19,30^. In this study, 5mC content and methylation level of wheat seedlings had tissue specificity and species specificity as found in *rice*^31^, and increased significantly in leaf along with the increase of water deficit, especially was significant in leaf of wheat AK58, but water deficit may cause the decrease of methylation level by up to 10% in *ryegrass*^13^. Under water deficit, the increase of DNA methylation took full-methylation as principle in wheat seedlings, estimating that the method of DNA methylation at CCGG sequence might be mainly double-strand full-methylation (CmCGG) in wheat seedlings.

It is well known, the status of DNA methylation would change in plant under adversity stresses, such as drought, low temperature, salt, etc^8,13,18,19^. Under drought stress, methylation status could change and methylation polymorphism may increase in *Dendrobium huoshanense* along with the increase of drought stress^32^, Hyper-methylation would occur in *Pisum sativum* and methylation level at second C in CCGG sequence might increase by 40%^20^. In this study, DNA methylation of wheat seedlings showed significant polymorphism under water deficit, such as methylation, hyper-methylation and demethylation, and these changes might take methylation as principle and demethylation as supplement. Some studies showed that methylation or demethylation in specific genetic loci might lead to different expression of gene in plant^33,34^, and the level of DNA methylation in gene is inversely related to its expression^35^. Therefore, wheat seedlings would potentially close or activate the expression of related genes by methylation or demethylation in response to water deficit.

In addition, the pattern of DNA methylation in different *rice* cultivars would change under drought stress^30,36^, it was also found that change of methylation pattern in two wheat cultivars was various along with the increase of water deficit, and was especially significant in wheat AK58, furthermore, the change of methylation pattern also exhibited various trends in different *potato* cultivars under drought stress^37^. Therefore, compared with that of wheat XM13, wheat AK58 might be more rapidly in response to water deficit by the change of DNA methylation.

## In conclusion

5mC content and methylation level of wheat seedlings exhibited tissue specificity, increased significantly in leaf along with the increase of water deficit, and the change of DNA methylation took methylation as principle and demethylation as supplement. Compared with that of wheat XM13, the change of methylation pattern in wheat AK58 was especially significant under water deficit, accompanied by higher methylation polymorphism, higher level of methylation and demethylation, perhaps wheat AK58 could be more rapidly in response to water deficit by the change of DNA methylation. Thus, this research would contribute to reveal molecular mechanism of wheat adapting to water deficit from the perspective of epigenetics, however it is still unclear how wheat would regulate the expression of related gene by changing DNA methylation in response to water deficit.

## Materials and methods

### Plant materials

In this study, wheat XM13 and wheat AK58 were used to be experimental materials, they were respectively cultivated by Xinxiang Academy of Agricultural Science and Henan Institute of Science and Technology, P. R. China. Compared to wheat XM13, the tolerance of wheat AK58 is extremely strong to drought, frost and disease, the yield of wheat AK58 is generally high and stable.

### Culture of wheat seedlings

Wheat seeds were firstly surface-sterilized for 10 min with 0.1% HgCl_2_, and were washed for 50 min by sterile water. Subsequently, wheat seeds were sown in pots equipped with nutrition soil, were cultured at 24 ± 1°C under 12 h photoperiod of 50 uE•m−2•s−1 light intensity, and were irrigated with 5 ml distilled water every two days. As cultured for 7 d, wheat seedlings were irrigated with 50 ml polyethylene glycol (PEG_6000_) solution every two days and had subjected to water deficit for 14 d.

In addition, according to the empirical equation derived by Michel and Kaufmann^38^, PEG_6000_ solution was prepared in this study, and six osmotic potentials of PEG_6000_ solution were respectively used, 0.00, −0.05, −0.15, −0.30, −0.50 and −0.75 MPa. Furthermore, about 50 wheat seedlings were treated in each stress group, there were three replicates per stress group. In this study, when wheat seedlings had been cultured for 21 d (three-leaf stage), genomic DNA methylation of wheat seedlings was analyzed.

### Extraction of genomic DNA

In this study, genomic DNA was extracted from leaf or root of wheat seedlings by cetyltriethyl ammonium bromide (CTAB) method^39^, the yield and purity of genomic DNA were detected at 260 nm by spectrophotometry. Moreover, the integrity of genomic DNA was determined with 0.8% agarose gel electrophoresis, it was found that this main band was very bright and free of tails. In addition, Genomic DNA was stored at −20 °C to analyze DNA methylation in leaf or root of wheat seedlings.

### Assay of 5mC content

The content of 5-methyl cytosine (5mC) in wheat genome was detected with HPLC as the method used by Duan *et al*.^40^. In this study, after about 10 μg genomic DNA was hydrolyzed with DNase I, nuclease P1 and alkaline phosphatase, was filtered with 0.45 μm microporous membrane and was detected with HPLC.

In this study, these conditions of HPLC were as follows, mobile phase was composed of 50 mM KH_2_PO_4_ and 8% methanol with pH 3.7 and 0.4 ml/min velocity, analytical column was Agilent C18 Zorbax XDB column (4.6 ×150 mm, 5 μm particle size), column temperature was 30 °C. Furthermore, according to the retention time of C and 5mC, non-methylated cytosine (C) and methylated cytosine (5mC) in genomic DNA could be detected with UV detector, detection wavelength was 285 nm, and the retention time of C and 5mC in genomic DNA of wheat seedlings was respectively 6.061 min and 6.896 min. In order to guarantee the reliability of experimental data, the precision, repeatability and stability of HPLC were tested, and the assay of 5mC content in genomic DNA of wheat seedlings was repeated three times.

### MSAP amplification

In this study, 9 primer combinations were screened from 40 primer combinations, the adapter, pre-amplification primer and selective amplification primer used in MSAP were all synthesized by Invitrogen (Beijing, China), these sequences of adapter and primer were listed in Table S1.

MSAP comprised enzymatic digestion reaction of *Eco*RI*/Hpa*II (H) and *Eco*RI*/Msp*I (M), the first enzymatic digestion reaction was conducted by 5 U *Eco*R I and 5 U *Msp* I, 5 U *Eco*R I and 5 U *Hpa*П were used in the second enzymatic digestion reaction. The reaction system of MSAP pre-amplification was 50 μl volume and was consisted of 75 ng *Eco*R I pre-amplification primer, 75 ng *Msp* I-*Hpa*П pre-amplification primer, 0.2 mM dNTPs, 1×PCR buffer, 1.5 mM MgCl_2_, 0.2 U Taq DNA polymerase, conditions of MSAP pre-amplification were listed in Table S2. The reaction system of MSAP selective amplification was 20 μl volume and was composed of 30 ng *EcoR* I selective amplification primer, 30 ng *Msp* I*-Hpa*П selective amplification primer, 0.2 mM dNTPs, 1 × PCR buffer, 2 mM MgCl_2_, 0.4 U Taq DNA polymerase. In addition, conditions of MSAP selective amplification were the same as these in MSAP pre-amplification except without Pre-PCR_1.

### MSAP data analysis

The level of genomic DNA methylation in wheat seedlings was quantified by MSAP binary data, the presence or absence of one band in MSAP was respectively scored as “1” and “0”, only clear and reproducible bands were scored after silver staining. Basing on the presence or absence of one band in H and M, genomic DNA methylation of wheat seedlings could be divided into three classes: the presence of band in H and M was considered to be non-methylation (class I), the presence of band in H and absence in M was considered as DNA hemi-methylation (class П), the presence of band in M and absence in H was considered to be DNA full-methylation (class III). Moreover, the level of DNA methylation was calculated by the following formulate: total DNA methylation level (%) = (bands of class П + bands of class III)/(bands of class I + bands of class П + bands of class III) × 100, hemi-metylation level (%) = bands of class II /(bands of class I + bands of class П + bands of class III) × 100, full-methylation level (%) = bands of class III/(bands of class I + bands of class П + bands of class III) × 100.

Compared with the control (CK), methylation patterns of genomic DNA in wheat seedlings were classified into polymorphism and monomorphism under water deficit. DNA methylation monomorphism (the status of band in M and H was same between CK and treatment group) was regarded to be Type A, DNA methylation polymorphism included Type B (DNA methylation) and Type C (DNA demethylation), thus changes of methylation status in wheat seedlings were divided into Type A, Type B and Type C under water deficit, and there were 12 kinds of band patterns (Table S3). The statistical formulates used to calculate polymorphism and monopolymorphism of DNA methylation were as follows: DNA methylation polymorphism (%) = (bands of Type B + bands of Type C)/(bands of Type A + bands of Type B + bands of Type C) ×100; DNA methylation monopolymorphism (%) = bands of Type A /(bands of Type A + bands of Type B + bands of Type C) ×100. In addition, some band patterns with indeterminable change of DNA methylation were also found in this study, but were not shown in these results.

### Statistics and analysis of data

In this study, significance level, analysis of variance (ANOVA) and multiple comparisons of Duncan’s multiple range test on the level of DNA methylation in wheat seedlings were performed with data processing system (DPS7.5), 5mC content in genomic DNA of wheat seedlings were calculated and analyzed by Excel and DPS7.5.

## Supplementary information

Supplementary information.
