# Supplementary material for: Responsive changes of DNA methylation in wheat (Triticum aestivum) under water deficit
Source: Sci Rep. 2020 May 13;10:7938. doi: 10.1038/s41598-020-64660-7 (PMC7220947; doi:10.1038/s41598-020-64660-7)
Supplement: Supplementary file 1 — Supplementary information. [file 41598_2020_64660_MOESM1_ESM.pdf]

Responsive changes of DNA methylation in wheat (*Triticum aestivum*) under water deficit

Hongying Duan, Jingyun Li, Yanqiu Zhu, Wenjing Jia, Huihui Wang, Lina Jiang, Yanqing Zhou*

Supplementary information

Table S1 The sequence of adapters and primers in MSAP

Table S2 The cycling conditions in pre-amplification of MSAP

Table S3 The pattern of DNA methylation in wheat seedlings under water deficit

Supplementary information

Table S1 The sequence of adapters and primers in MSAP

No.	Adapter and primer	Sequence
1	*Eco*RI adapter	5’-CTCGTAGACTGCGTACC
		CTGACGCATGGTTAA-5’
2	*Msp*I and *Hpa*II adapter	5’-GACGATGAGTCTAGAA
		CTACTCAGATCTTGC-5’
3	*Msp*I and *Hpa*II adapter	5’- GATCATGAGTCCTGCT
		GCTCGTCCTAGTACT-5’
4	*Eco*RI pre-amplification primer	5’-GACTGCCTACCAATTCA-3’
5	*Msp*I/*Hpa*II pre-amplification primer	5’-GATGAGTCTAGAACGGT-3’
6	Selective amplification primer	Eo: 5’-GACTGCCTACCAATTC
		MHo: 5’-GATGAGTCTAGAACGGT
7	*EcoR*I selective amplification primer	E1:Eo-AAC E2:Eo-AAG E3:Eo-ACA
		E4:Eo-ACT E5:Eo-AGG E6:Eo-ACC
		E7:Eo-ACG E8:Eo-AGC
8	*Msp*I/*Hpa*II selective amplification primer	MH4: MHo-ACA MH5: MHo-ACC
		MH6: MHo-CCG MH7: MHo-ACT
		MH8: MHo-TAA

Table S2 The cycling conditions in pre-amplification of MSAP

Cycle name	Temperature (℃)	Time	Number of cycles
Pre-PCR_1	72.0	1m	1
Pre-PCR_2	94.0	45s	
	65.0	30s	1
	72.0	1m	
Touch-down PCR	94.0	30s	
	64.3	30s	12
	72.0	1m	
PCR	94.0	30s	
	55.9	30s	20
	72.0	1m	
Elongation	72.0	30s	1
Store	4.0	-	-

The annealing temperature of Touch-down PCR was respectively 64.3℃, 63.6℃, 62.9℃, 62.2℃, 61.5℃, 60.8℃, 60.1℃, 59.4℃, 58.7℃, 58.0℃, 57.3℃ and 56.6℃, respectively.

Table S3 The pattern of DNA methylation in wheat seedlings under water deficit

No.	Band				Change of methylation pattern		Type of methylation pattern	
	H1	M1	Hx	Mx	CK	Treatment		
1	1	1	1	1	CCGG GGCC	CCGG GGCC	A1	
2	1	0	1	0	CCGG CCGG GGCC GGCC	CCGG CCGG GGCC GGCC	A2	A
3	0	1	0	1	CCGG GGCC	CCGG GGCC	A3	
4	1	1	1	0	CCGG GGCC	CCGG CCGG GGCC GGCC	B1	
5	1	1	0	1	CCGG GGCC	CCGG GGCC	B2	
6	0	1	0	0	CCGG GGCC	CCGG GGCC	B3	B
7	1	0	0	0	CCGG CCGG GGCC GGCC	CCGG GGCC	B4	
8	1	1	0	0	CCGG GGCC	CCGG GGCC	B5	
9	0	1	1	1	CCGG GGCC	CCGG GGCC	C1	
10	1	0	1	1	CCGG CCGG GGCC GGCC	CCGG GGCC	C2	
11	0	0	0	1	CCGG GGCC	CCGG GGCC	C3	C
12	0	0	1	1	CCGG GGCC	CCGG GGCC	C4	

H and M represented *Eco*RI/*Hpa*II (H) and *Eco*RI/*Msp*I (M), H1 and M1: bands amplified from genome of wheat seedlings under the normal condition, Hx and Mx: bands amplified from genome of wheat seedling treated with PEG_{6000} solution. Scores of 1 and 0 indicated the presence or absence of bands in MSAP, “1 1”, “1 0” and “0 1” was considered to be the pattern of non-methylation, hemi-methylation or full-methylation, respectively.
